# Reverse innovation: an opportunity for strengthening health systems

**DOI:** 10.1186/s12992-015-0088-x

**Published:** 2015-02-07

**Authors:** Anne W Snowdon, Harpreet Bassi, Andrew D Scarffe, Alexander D Smith

**Affiliations:** Ivey International Centre for Health Innovation (ICHI), Ivey Business School at Western University, 1255 Western Road, London, Ontario N6G 0 N1 Canada; Faculty of Health and Rehabilitation Sciences, Western University, 1151 Richmond Street, London, Ontario N6A 3K7 Canada

**Keywords:** Reverse innovation, Innovation, Health, Health systems, Costs, Leadership development, Competition

## Abstract

**Background:**

Canada, when compared to other OECD countries, ranks poorly with respect to innovation and innovation adoption while struggling with increasing health system costs. As a result of its failure to innovate, the Canadian health system will struggle to meet the needs and demands of both current and future populations. The purpose of this initiative was to explore if a competition-based reverse innovation challenge could mobilize and stimulate current and future leaders to identify and lead potential reverse innovation projects that address health system challenges in Canada.

**Methods:**

An open call for applications took place over a 4-month period. Applicants were enticed to submit to the competition with a $50,000 prize for the top submission to finance their project. Leaders from a wide cross-section of sectors collectively developed evaluation criteria and graded the submissions. The criteria evaluated: proof of concept, potential value, financial impact, feasibility, and scalability as well as the use of prize money and innovation team.

**Results:**

The competition received 12 submissions from across Canada that identified potential reverse innovations from 18 unique geographical locations that were considered developing and/or emerging markets. The various submissions addressed health system challenges relating to education, mobile health, aboriginal health, immigrant health, seniors health and women’s health and wellness. Of the original 12 submissions, 5 finalists were chosen and publically profiled, and 1 was chosen to receive the top prize.

**Conclusions:**

The results of this initiative demonstrate that a competition that is targeted to reverse innovation does have the potential to mobilize and stimulate leaders to identify reverse innovations that have the potential for system level impact. The competition also provided important insights into the capacity of Canadian students, health care providers, entrepreneurs, and innovators to propose and implement reverse innovation in the context of the Canadian health system.

## Background

### Canada’s innovation track record

Every developed country in the world is challenged by the increasing demands for health services and the rising costs of health care that are closely associated with rapid advances in technology and aging populations. Canada is no exception; it is facing similar challenges, yet has made less progress in meeting these demands when compared to other developed countries. Within the context of the Canadian economy, health care spending totals $207 billion, representing 11.6% of the national gross domestic product [1]. As demand continues to outpace the capacity to deliver health services, approaches that embrace new technologies and leverage innovative processes will become pivotal to the sustainability of health systems both within Canada and globally. In Canada, a slow uptake and adoption of innovative technologies, processes, and procedures is leading to growing cost pressures on Canada’s publicly funded health systems which continue to be challenged to deliver high quality care and value to Canadians [2].

Canada leads the world in its population percentage with completed tertiary education (51%), inclusive of both undergraduate and graduate degrees [3]. Canada is also among the top four countries in the world to produce new knowledge (i.e. publications, patents, technologies) [4]. Yet, despite a social framework that supports and encourages Canadians to pursue advanced education and new knowledge, Canada receives failing grades when it comes to their ability to adopt innovation. According to McKinsey & Company, Canada placed 13^th^ out of 17, earning a “D” grade, among developed nations in this economic and future prosperity indicator [2,5]. The economic and future prosperity indicator ranking indicates that as a proportion of its overall economic activity and in comparison to other OECD countries, Canada does not rely on innovation as much as some of its peer comparators [2]. Frequently Canada is viewed a country of pilot projects that are not scaled or adopted across health systems to achieve financially sustainable programs with learnings and/or outcomes that are translated and shared across provincial or national jurisdictions [6].

### Opportunity for reverse innovation

Health systems require constant innovation to control ballooning costs, to create new knowledge as well as develop more effective and efficient mechanisms of achieving value for Canadians. To accelerate the adoption and scalability of successful innovation and thereby support Canadian health systems to overcome the limitations of pilot programs, reverse innovation can offer Canada opportunities to learn from innovations that are occurring in developing and/or emerging markets and consider how these innovations may be translated into the context of the Canadian health system. These reverse innovations are an important opportunity for learning and building capacity for leading change that has the potential to optimize resource use while also finding innovative approaches to deliver health services in a cost effective, sustainable manner. The purpose of this project was to examine the potential for reverse innovation to offer Canadian health systems a new approach to supporting and strengthening innovation adoption by learning from global health systems. This manuscript documents the use of a competition approach to encourage Canadians to look beyond national boarders, to developing and emerging markets, for innovations that can address current Canadian health system challenges. In an effort to better understand how the concept of reverse innovation could be mobilized amongst Canadians, the Ivey International Centre for Health Innovation issued an open call to invite proposals for reverse innovations that could address Canada’s health system challenges. One of the key objectives of this initiative was to strengthen leadership capacity among future leaders to identify and lead potential reverse innovation projects that address health system challenges in Canada. A growing base of research indicates that a large proportion of cost and value-based innovations are coming from emerging markets – an opportunity for learning which is not evident in the work of Canadian innovators or health systems [7].

As health systems, in particular those of developed nations, quickly consume the current financial resources to maintain delivery of high quality care, the concept of “doing more with less” or “innovation by necessity” plays a prominent role in the future of these health systems. Canada’s limited success in health system innovation is believed to be related to several barriers, including:

1. **Lack of Competition:** In many countries with privatized service delivery, private sector companies are forced to innovate into the mainstream market, or they will face erosion of existing market position in the world. This incentive for individual companies to innovate is not seen to the same extent in publicly funded systems like in Canada, where there is a lack of competition [8].
2. **Collaboration Challenges:** In developed nations with complex and long standing bureaucratic legacy systems, collaboration is often difficult due to the complexity and number of stakeholders in each health system. In developing countries, the drive for competition coupled with fewer (or less complex) bureaucratic systems, the potential for collaboration is may be more easily overcome.
3. **Change-Resistant Culture:** Canada is not known as a culture that embraces change and where entrepreneurship thrives. Innovators are often met by a change-resistant culture from the necessary stakeholders [9].
4. **Need for Change:** While Canada maintains an international reputation for delivering high quality health services with positive patient outcomes, it presents an increasing financial burden on Canadian tax payers. Important lessons can be learned from emerging markets with less developed health systems that achieve positive patient outcomes with fewer resources. The idea of “doing more with less” is a lesson that presents tremendous opportunity to increase the financial sustainability of the Canadian health system.

Reverse innovation, the two part process whereby innovations are designed and created for emerging markets, and then brought to developed nations, has the potential to positively affect financially burdened, developed health systems through quality innovations at decreased costs [7]. Although Canada could benefit substantially from reverse innovation, we have not been successful at implementing these innovations into the mainstream market. Canada needs to build capacity for learning from developing countries, translating innovations that are occurring into the Canadian context to achieve value for Canadians and sustainability for health systems. For example, GE Healthcare Worldwide has created a portable, durable, rechargeable and low-cost electrocardiogram (ECG) (i.e. MAC i) that was designed to service rural and low-income areas of India [10,11]. Other examples of reverse innovations that have had application to healthcare include crowdsourcing programs that were developed to map disaster impact and response to earthquakes as well as service delivery models that have addressed geographic barriers that restrict access to conventional medical infrastructure in rural Haiti [10]. Reverse innovations, like those created by GE Healthcare and those programs and models developed in Haiti, have the potential to positively affect developed health systems through quality innovations that achieve value at decreased costs.

Currently the concept of reverse innovation is emerging as a potential solution for over burdened, financially constrained health systems [10]. However, the capacity for Canadians to identify and adopt reverse innovations into the Canadian context has not been explored to date. As such there is a need to mobilize Canadian capacity to identify reverse innovations that have the potential to positively impact health systems. The *Reverse Innovation Challenge,* a cross-sector competition which invites participants to identify and test reverse innovations across the globe to address Canadian health system challenges.

### Project goal:

*The goal of our project was to examine if a reverse innovation competition can mobilize capacity among future leaders to identify potential reverse innovation projects that address health system challenges in Canada*.

For the purposes of this project, mobilizing capacity for reverse innovation was viewed with the intent of facilitating an environment, for future and current health system leaders, to engage in meaningful, constructive dialogue surrounding the potential for reverse innovation within the Canadian health system. The competition not only created an opportunity for health system leaders to engage the concept of reverse innovation, but also the greater population at large was engaged through different media and news outlets successfully generating awareness of the concept of reverse innovation in Canada.

### Approach to the project

#### Why a competition?

Due to the emerging evidence of the value of reverse innovation in global communities, the concept was examined in the Canadian context through an open competition. Taking an exploratory approach to the construct of the competition would enable any interested parties to participate and also enable an assessment of how reverse innovation is perceived. The challenge was designed to create an environment that encouraged Canadians to look beyond national borders, identify potential reverse innovation opportunities in emerging and developing markets, and consider how those innovations may address challenges within the Canadian health system.

The format of the competition was based on an environmental scan of successful competitions, including discussions with organizations with experience hosting similar competitions such as: Ashoka Change Makers, Health Council of Canada and Grand Challenges Canada, including the provision of a monetary prize to advance the winning idea from concept to testing in the Canadian health care context. The competition was financially supported by a number of partners including: Medtronic Canada, GE Healthcare, Telus Health, Sandra Rotman Centre, Roche Canada and the Institute for Health System Transformation and Sustainability. Representatives from each of these organizations formed an advisory group which informed the planning, structure, format and process throughout the duration of the competition.

Further, the concept of the competition was shared and tested with various experts, beyond the advisory group and who are considered experts within the fields of innovation and global health, for feedback and input. External experts from multiple perspectives were engaged in guiding the process, including: academics, researchers, clinicians, granting organizations, health system leaders and industry leaders. All of the stakeholder comments were taken into consideration and the concept was refined accordingly. Based on the feedback and recommendations, the evaluation criteria was revised to ensure it addressed feasibility, scalability and weighted more heavily projects with partnerships in emerging markets. The evaluation criterion is discussed in more detail below.

#### Building momentum through communication and awareness

The competition invited the public to identify innovations from emerging markets that could be applicable in the Canadian context to tackle some of our greatest health care challenges. While the competition was open to the general public, the communication strategy focused largely on students, international innovators and entrepreneurs, health system leaders, and care providers. The communications plan, detailed below, identified strategies and tactics targeted towards each of these groups.

Over the course of the four months, the Centre communicated information about the competition via: a dedicated website page promoting the challenge; targeted email outreach to various distribution lists including research institutions and universities across Canada; social media updates via Facebook and Twitter (#richallenge), including two online chats. Sponsors of the competition also promoted the Challenge through their communications channels; academic and professional speaking engagements where information about the Challenge was provided; and through the Canadian College of Health Leaders network, MEDEC and Rx&D communications vehicles. In addition, the competition launch was supported by two media outlets: Makeshift Magazine and Rogers Healthcare Innovation and Research Magazine. Both media outlets actively promoted the competition through their print and online channels.

### Evaluation criteria and committee

The approach to adjudicating proposals was based on the two-step process that focused on two central criteria: (1) feasibility of the proposed emerging market design and (2) translation of how the identified reverse innovation is proposed to be adopted into a developed health system [7]. The evaluation criteria were designed to ensure that both of these elements were appropriately addressed and captured in the review process. One of our key assumptions was that it was highly possible that there would be no submissions where the reverse innovation identified had successfully been applied or adopted in the Canadian context. Therefore, the criteria required the proposed innovation must be one that had been implemented and had demonstrated evidence of its potential for adoption and scalability. Seven criteria were designed to help assess the submissions (See Table 1).

**Table 1:** **Evaluation criteria**

**Criterion**	**Description**	**Value as a % of overall score**
1. Demonstrated Proof of Concept	Was there documented evidence that the innovation achieved impact in an emerging market/developing country context? For the purpose of the evaluation the notion of “documented” was considered broadly and included grey literature.	10%
2. Potential Value to Canadian Health Systems	Would the proposed innovation achieve value if applied in Canada and if so, would it deliver value - better outcomes or reduced cost, or both - if adopted in a health system context?	25%
3. Economic Impact if Implemented	What would be the economic impact for the Canadian economy if the innovation were to be implemented? Recognizing that this is potentially difficult to measure, for the purpose of the competition, this criterion was intended to ensure applicants considered broader technological, societal, economic and political considerations associated with the innovation.	10%
4. Feasibility	Did the applicants identify barriers, risks, challenges and enablers/strengths associated with the innovation and its adoption in the Canadian context and did they provide and strategies to either mitigate risks or leverage strengths?	15%
5. Potential for Scalability	Is there a clear path identified for scaling up the innovation across health systems, beyond a pilot project, at a broader system level? If so, what conditions, such as policy, reimbursement, education, culture, data, technology etc., need to be in place to scale the innovation.	25%
6. Use of Prize Money	How was the proposed budget going to be used to advance the proposed reverse innovation? This criterion was seeking a creative yet realistic use of fund and also served as a mechanism for accountability and transparency for sponsors of the competition.	5%
7. Innovation Team	The degree to which the team assembled represents key stakeholders in health systems (clinicians, industry leaders, policy makers, Canadians) to drive the adoption of the innovation. This criterion was aimed to drive partnership, collaboration and sharing of learnings across jurisdictions. Applicants with partnerships in emerging markets where the concept was developed and tested were weighted more heavily to stress the importance of and attempt to facilitate bi-directional learning.	10%

The applicants were required to submit a two-minute video that would describe the concept succinctly and in a clear and easily understood language. Finally, each submission required a Canadian project team leader to increase the likelihood of successful implementation and adoption in a Canadian health system.

Given the limited body of research describing reverse innovation in health care, and the novelty of the Reverse Innovation Challenge, an evaluation committee was established to evaluate the submission. The committee members we selected based on their experience and skills to ensure a broad range of perspectives were represented. Each evaluator was assigned a primary and secondary submission to review; assignments were based on the expertise and experience of the reviewer and the fit with the application. The evaluation committee then convened in a daylong session to review the submission videos, hear feedback from the primary and secondary reviewers for each application and then discuss each proposal. The criteria were used to score each submission. Each application was reviewed by the committee using the criteria to score each submission. Each application was assigned two reviewers, scores and reviews were discussed, after deliberate discussion and consideration, the committee reached consensus on the score for each application. Once the committee scored all of the proposals, the proposals were ranked and a short list of the top five proposals was determined and then further discussed and ranked to identify the top three proposals and finally the highest ranked proposal.

### Outcomes

The Centre officially launched Colour Outside the Lines: A Reverse Innovation Challenge in Canadian Health Systems in January 18, 2013. The competition closed on May 31st, 2013. The winners of the competition were announced at the Reverse Innovation in Health Care Conference in November 2013.

During the four-month submission process, the Centre received a total of 12 submissions. A list of the projects can be found below in Table 2. Key highlights from the submissions included:

**Table 2:** **Summary of competition submissions**

**Proposal**	**Type of innovation**	**Emerging market where innovation has been applied**	**Summary**
1)	Technology	India	An effective, non-expert operable, non-invasive cardiovascular screening tool to be used by general medical practitioners.
2)	Process	Singapore & Japan	Through the means of an app, the patient and their families would be able to centralize all administrative tasks of a hospital visit based on their services required and receive responses.
3)	Process	Thailand, Bangladesh, Pakistan, Ethiopia, & Tanzania	Use of a community health worker (CHW) model who handle routine tasks in primary care, where intensive follow-up is needed to ensure patients follow the best treatments.
4)	Education	India, China, South Africa	Development of a set of cross-cultural educational and communication tools for menopausal women of differing ethnicities to improve communications between women and their caregivers.
5)	Process	Thailand	Decreasing isolation to improve mental wellness while promoting healthy eating habits, and encouraging volunteerism for seniors.
6)	Technology	Kenya & Bangladesh	Biometric authentication of ID and key health information, and access health records or caregiving requirements via data or even SMS.
7)	Technology/mHealth	Malawi	Apply the innovative use of radio and new information and communication technologies—mobile phones, podcasting and cloud-based interactive voice response services—for health promotion.
8)	Technology	N/A	The use of red lights at night in hospital rooms to permit the secretion of melatonin and help keeping a good sleep-wake cycle.
9)	Technology	India	A non-mydriatic eye prescreening tool which can detect glaucoma, diabetic retinopathy and corneal disease with high sensitivity, and minimal invasiveness.
10)	Process	India, South Africa & Pakistan	A pictogram-based toolkit to improve wayfinding on hospital campuses, and a simplified discharge summary designed with and for patients to improve the comprehension of medication and care instructions.
11)	mHealth	N/A	mHealth solution to the underuse of radiotherapy in end-of-life cancer patients.
12)	Technology/mHealth	Southern and southwestern Asia, Tanzania, Uganda, and Ghana, Serbia, Peru	The use of mobile technology or m-health to transfer information to new mothers of late preterm infants, with focus on health promotion to improve efficiency in care following discharge from hospital.

- Four of the submissions were focused on mHealth and leveraged the use of smartphones and mobile apps, two were devices, and three of them were related to education and communications focused on health issues.
- Six of the projects focused on immigrant or aboriginal populations. One of them focused on seniors and two on women’s health and wellness.
- The backgrounds of the applicants ranged from undergraduate students, medical students, clinicians, academics and entrepreneurs.

The literature describing reverse innovation presents varied definitions of reverse innovation. The committee engaged in a robust debate and discussion about the definition of reverse innovation and how the applicants were applying it to the Canadian health system. The evaluation committee was comprised of members with a wide range of expertise and leadership experience; all evaluation committee members were considered experts in the field(s) of health systems, innovation and/or reverse innovation. The expertise of the evaluation committee members varied from: information technology (IT), medical devices, finance, international/global health, rural health, aboriginal health, gender/racial health and social enterprise. As a result of the wide array of experiences and interpretations of reverse innovation, the dialogue amongst the evaluation committee lead to a constructive debate pertaining to the value proposition affiliated with reverse innovation. The scoring of the individual competition submissions was varied and was reflective of the different value perspectives of the committee members. Ultimately, differences were resolved through lengthy discussions and consensus agreement between committee members.

The top five submissions were short-listed and two submissions were identified as not meeting the criteria of reverse innovation. The top 5 submissions were able to demonstrate strong proof of concept and value by effectively defining how the innovation would achieve value for the Canadian health system. To varying degrees, the top five submissions also acknowledged and engaged relevant partnerships for successful implementation and potential for scalability. The five finalists were then asked to submit some additional information to provide further clarity on their proposals to the committee. The additional information requests included:

- *Further development and discussion of key component of the proposal;*
- *Further consideration to potential challenges with adoption in Canada including; stakeholder engagement and how these challenges might be mitigated;*
- *Considerations for how the project might be scaled beyond a pilot in Canada;*
- *Updates on any progress or work on the project since submission.*

The five finalists were first informed that they were short-listed and then were asked for permission to profile their application online to engage comments and feedback from the public online. Their proposals were posted on the website and comments were welcomed from the public for a one month period. The intention was that evaluation committee would use the public comments aligned with the additional information to inform their final decision. However, due to technical challenges with the website, the comments were not considered in the evaluation process.

The committee reconvened for a second stage evaluation, to evaluate each short listed application to consider the strengths of the additional information provided to the committee. Once again, ranking of the proposals was reviewed and determined based on consensus of the committee. The committee unanimously agreed on the winner of the challenge. The committee further determined that only one submission met the requirements and spirit of the competition (#9); this team was awarded financial support and mentorship to trial their reverse innovation in the Canadian health system context. The decision to award only one prize was made with careful and deliberate consideration by the evaluation committee, based solely on the follow up information provided by each of the top five teams. The winning team was announced on November 26, 2013.

## Discussion

The International Centre for Health Innovation at the Ivey Business School at Western University is now working with the winning team on a demonstration project to support implementation of the winning reverse innovation and will help assess whether its application is feasible in the Canadian context. The winning team will report progress over the course of the next 18 months.

Overall, the competition proved to be a successful mechanism to mobilize the capacity of future leaders to identify potential reverse innovation projects that address Canadian health system challenges. The competition facilitated constructive dialogue and debate on the subject of reverse innovation between health system stakeholders; inclusive of stakeholders from: health service delivery (e.g. clinicians, etc.), government (i.e. provincial & federal), private industry (e.g. IT, medical devices, etc.) and academia. The challenge also leveraged social media (e.g. Facebook, Twitter, YouTube etc.), more tradition forms of media (e.g. magazines and newspapers), as well as academic conferences and symposia to engage the global community in an effort to mobilize the capacity of current and future health system leaders; an effort that established reach to a minimum of 400,000 individuals. While recognizing that the intention of the reverse innovation competition was to mobilize capacity to identify reverse innovation projects, it is important to acknowledge that, as a result of their participation in and profile received from the reverse innovation competition, three reverse innovation projects received funding to trial their reverse innovations within the Canadian health system. One project received funding directly from the prize allocated to the competition and two other projects received government funding.

Recognizing that this entire project was an innovation unto itself and a learning experience, there were a number of challenges with the administration of competition itself. For example, the website was entirely developed and maintained by a volunteer student team. Due to technical challenges, the process for the second stage evaluation did not use those public comments to inform their decision as it was intended to.

Another limitation was the novel nature of the concept (applying reverse innovation in a Canadian context), as there was no existing baseline knowledge or experience for reverse innovation ideation or evaluation in Canadian health systems, from which the evaluation committee could assess the submissions. Therefore, the definition applied for the purpose of the challenge left significant room for interpretation. Further, the evaluation rubric, adapted from the Centre’s framework for innovation, had not been tested in this context.

The experience of creating a reverse innovation challenge offered a significant learning opportunity for the Ivey International Centre for Health Innovation. It also raised the profile of reverse innovation discussion and profile across the country, and provided important insights into the capacity of Canadian students, healthcare providers, entrepreneurs, and innovators to propose and implement reverse innovation in the context of Canadian health systems.

## Conclusion

The Colour Outside the Lines: A Reverse Innovation Competition successfully stimulated future leaders to identify and lead projects that were driven by reverse innovations from emerging and developing markets. The winning submission of this competition will use the prize money to implement their reverse innovation in the Canadian health system and will report on their progress over the next 18 months.
